# Supplementary figures and images for: Multi-epitope vaccine design against Monkeypox virus: An immunoinformatics approach
Source: PLoS One. 2026 Feb 13;21(2):e0342087. doi: 10.1371/journal.pone.0342087 (PMC12904572; doi:10.1371/journal.pone.0342087)

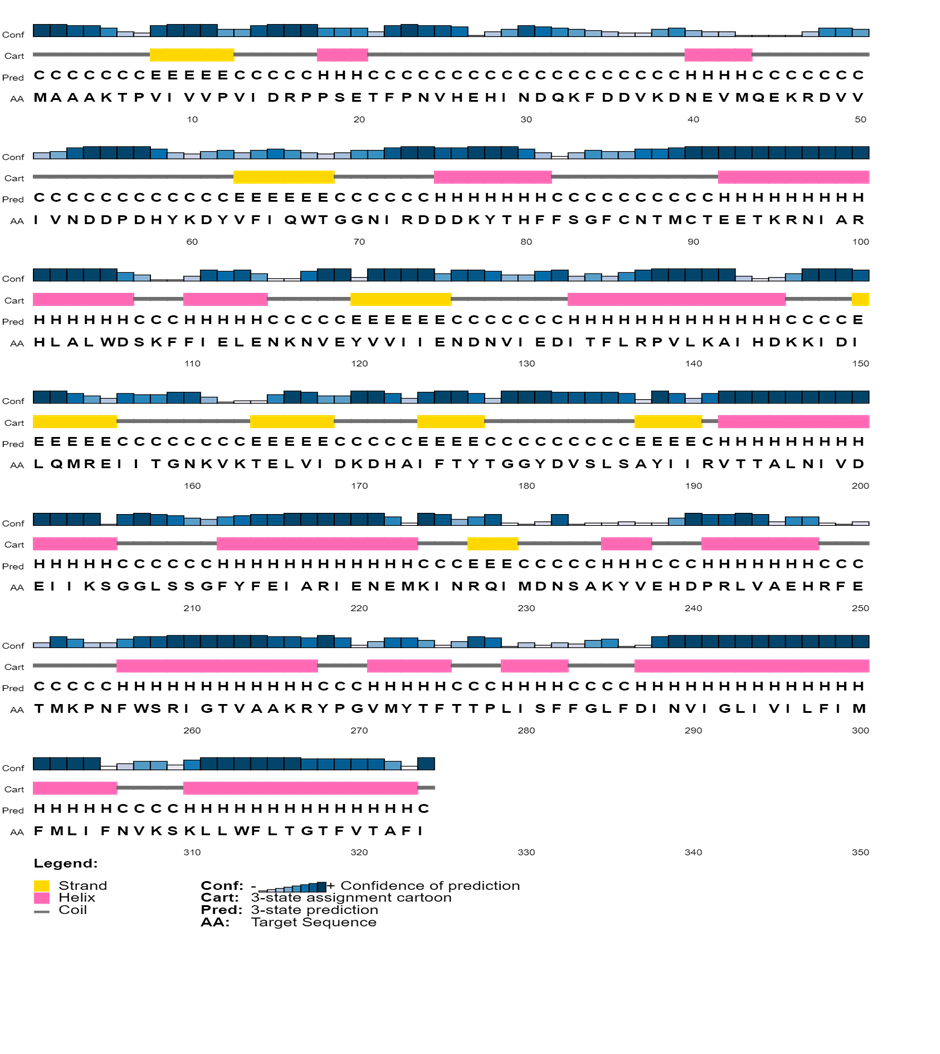

Supplement: S1 Fig — (DOCX) [file pone.0342087.s001.docx]

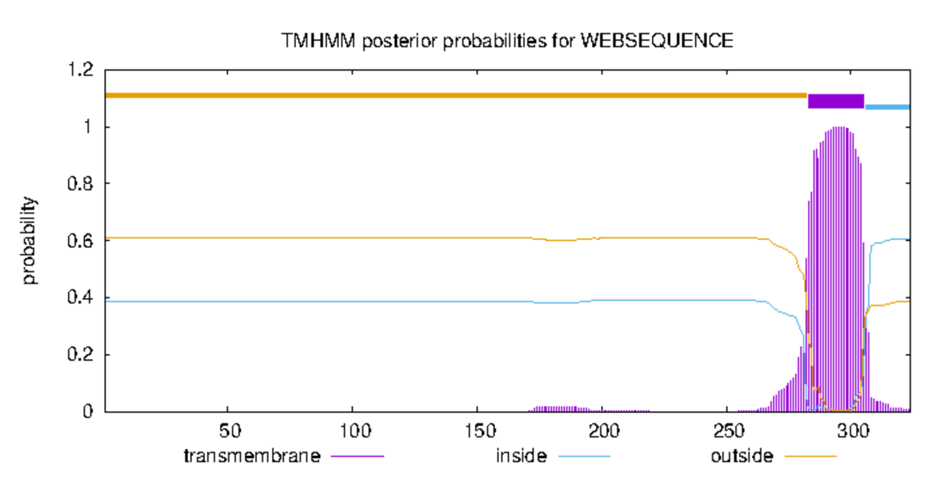

Supplement: S2 Fig — (DOCX) [file pone.0342087.s002.docx]

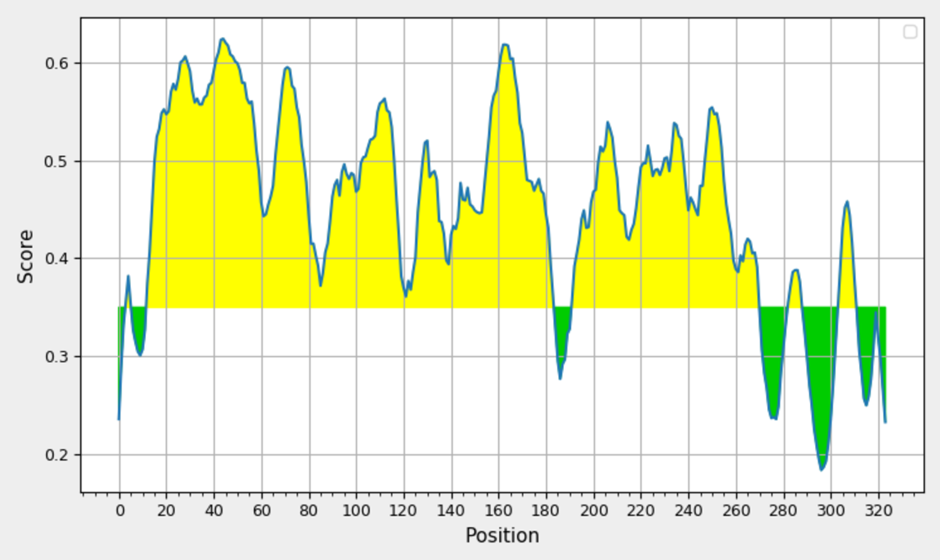

Supplement: S3 Fig — Threshold line: anything above it is predicted as an epitope. (DOCX) [file pone.0342087.s003.docx]

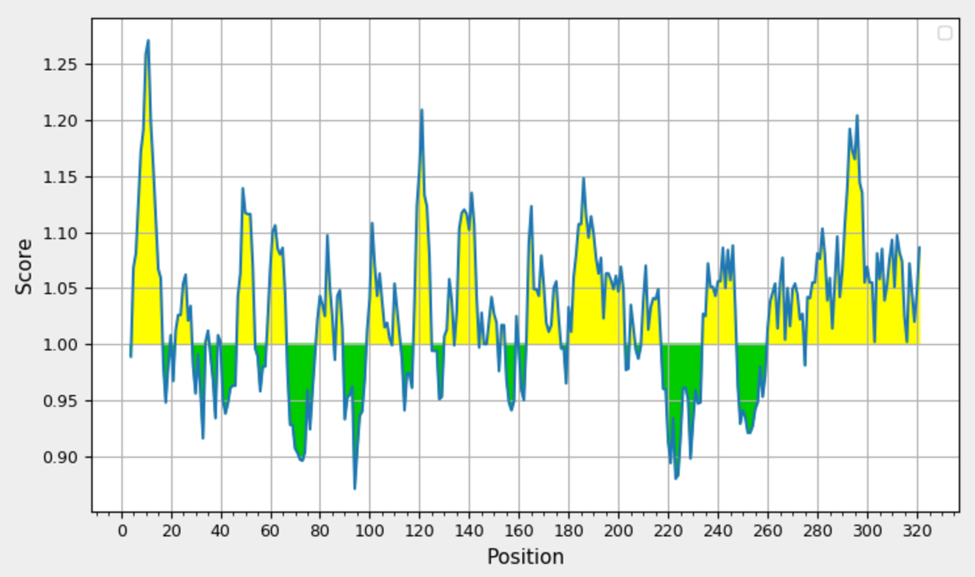

Supplement: S4 Fig — (DOCX) [file pone.0342087.s004.docx]

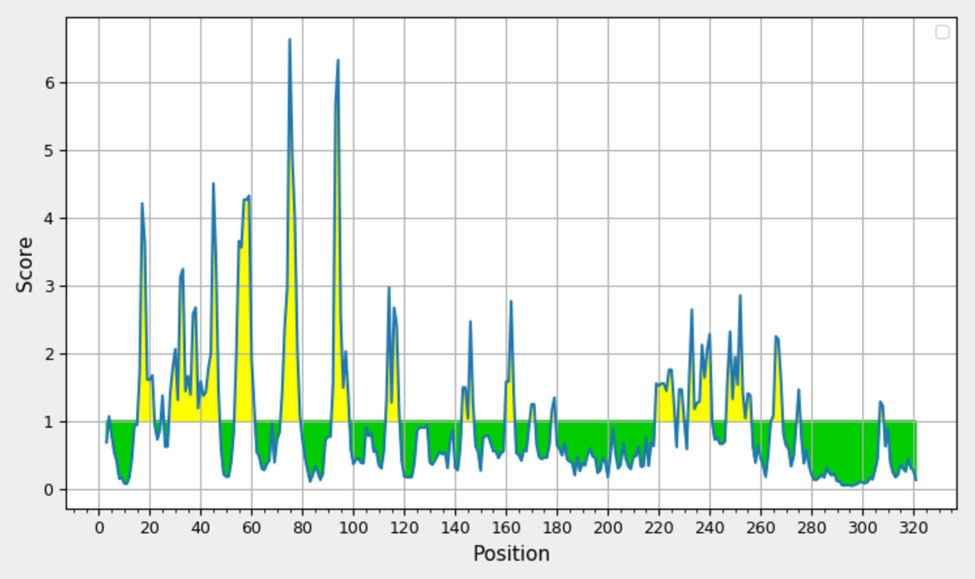

Supplement: S5 Fig — (DOCX) [file pone.0342087.s005.docx]

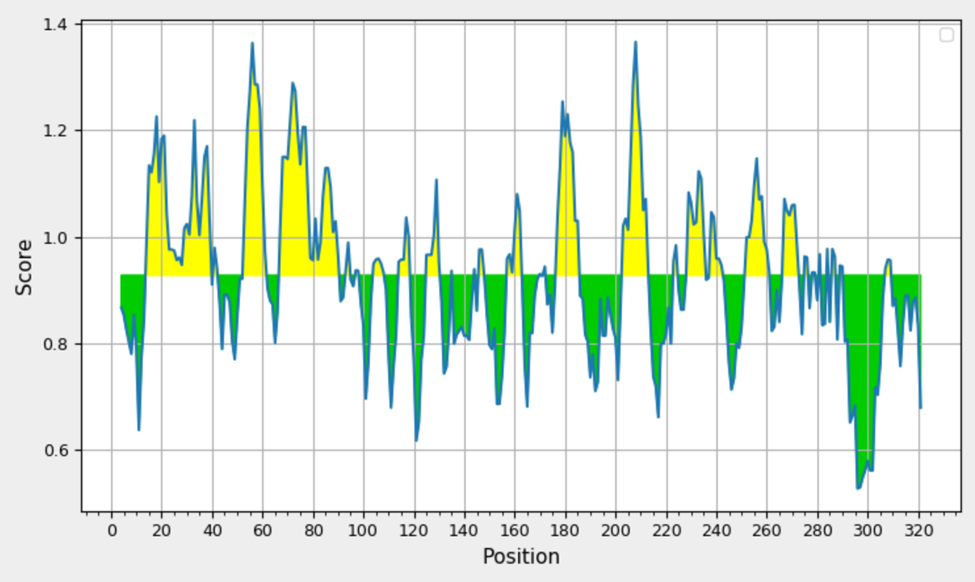

Supplement: S6 Fig — (DOCX) [file pone.0342087.s006.docx]

**
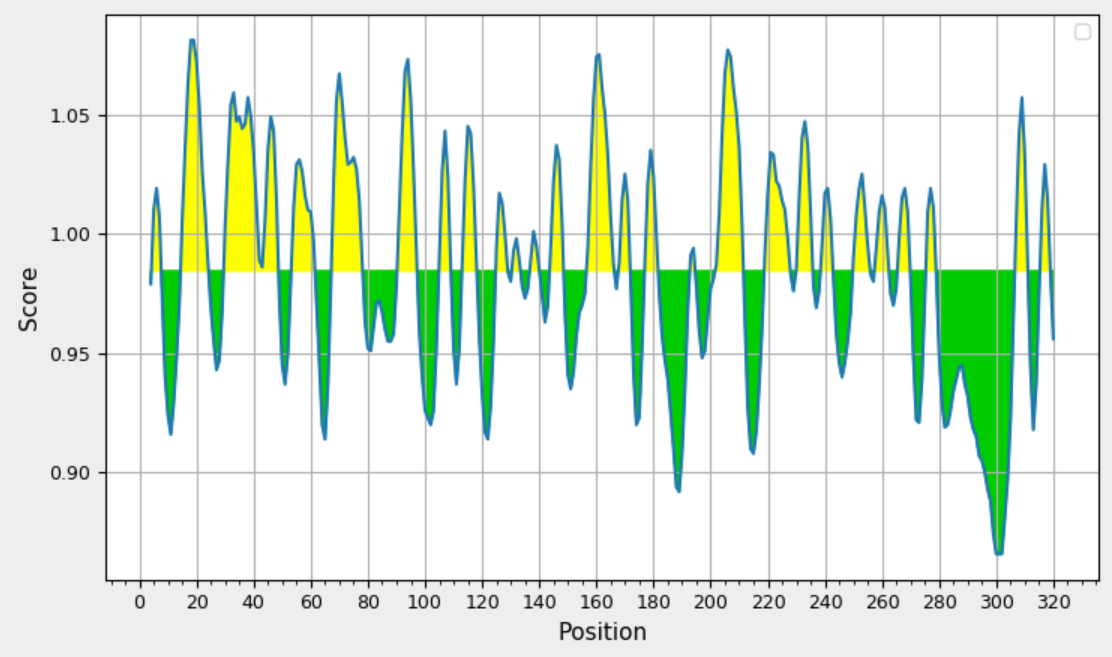
**

Supplement: S7 Fig — (DOCX) [file pone.0342087.s007.docx]

**
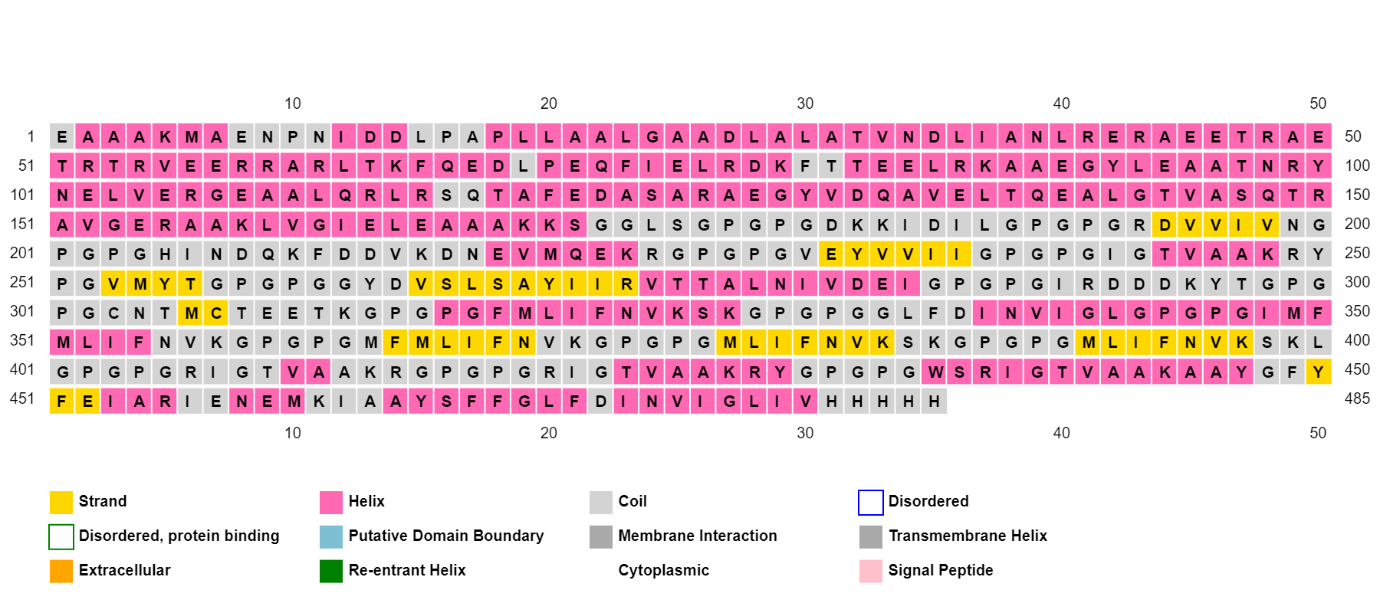
**

Supplement: S9 Fig — (DOCX) [file pone.0342087.s009.docx]

**
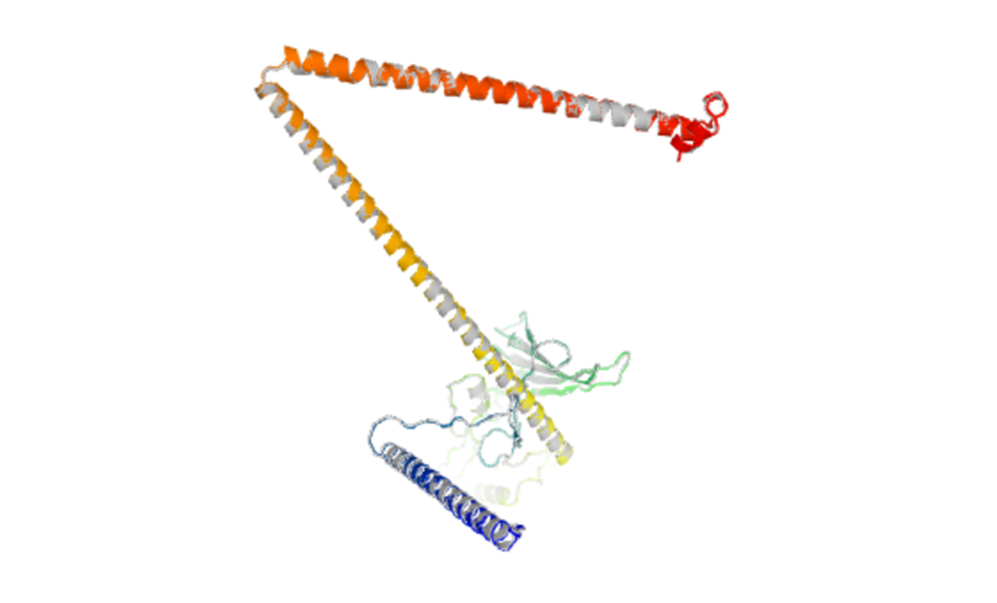
**

Supplement: S10 Fig — (DOCX) [file pone.0342087.s010.docx]

**
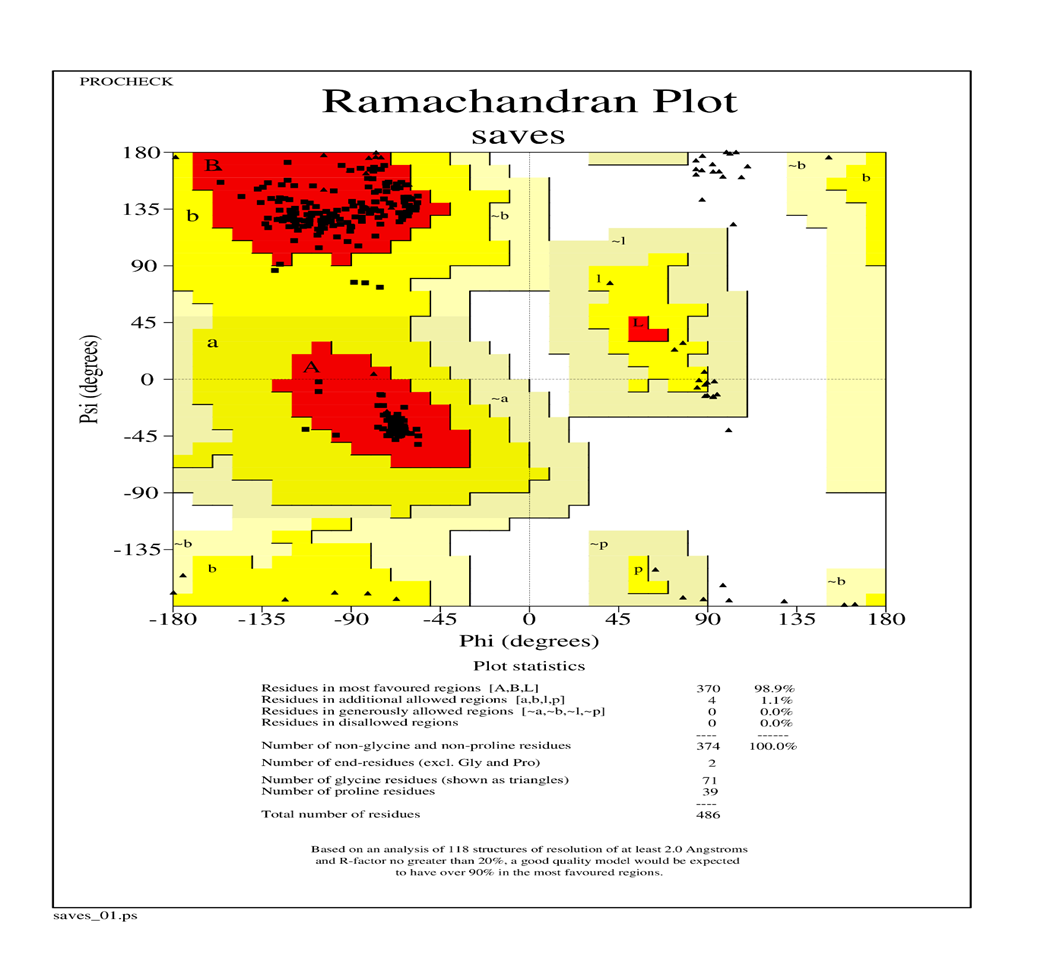
**

Supplement: S11 Fig — (DOCX) [file pone.0342087.s011.docx]

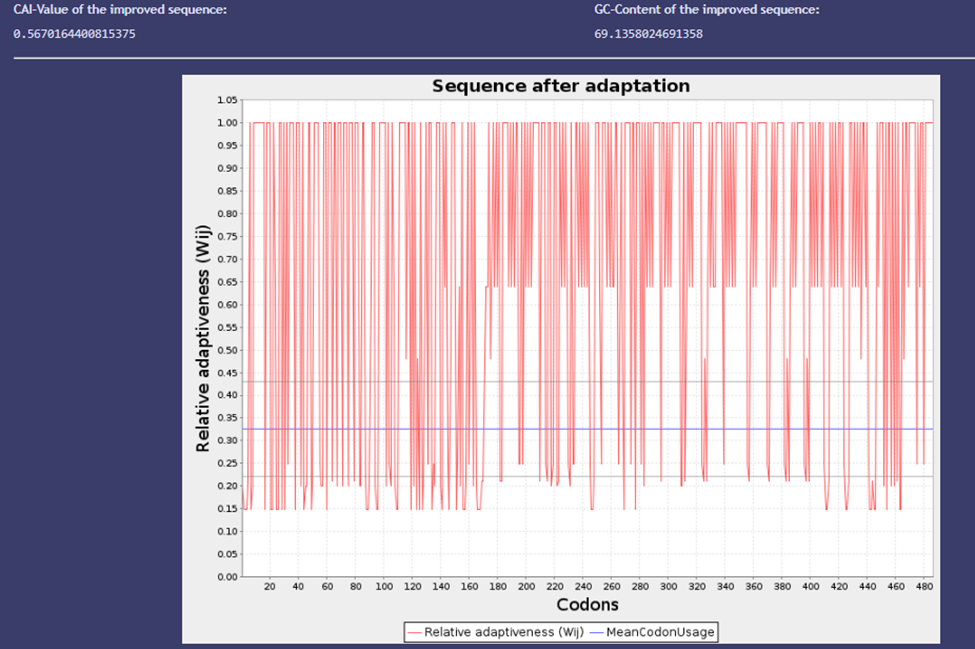

Supplement: S12 Fig — (DOCX) [file pone.0342087.s012.docx]
