# Supplementary material for: Multi-epitope vaccine design against Monkeypox virus: An immunoinformatics approach
Source: PLoS One. 2026 Feb 13;21(2):e0342087. doi: 10.1371/journal.pone.0342087 (PMC12904572; doi:10.1371/journal.pone.0342087)
Supplement: S1 Table — (DOCX) [file pone.0342087.s013.docx]

| **Peptide** | **Antigenicity** | **Allergenicity** | **Toxicity** | **Allele** | **Start** | **end** | **length** | **ic50** | **rank** | **Conservancy %** |
| --- | --- | --- | --- | --- | --- | --- | --- | --- | --- | --- |
| CNTMCTEETK | (1.2298) Antigen | Non-Allergen | Non-Toxic | HLA-A*68:01 | 16 | 25 | 10 | 58.24 | 0.5 | 100% |
| FMLIFNVKSK | (1.6619) Antigen | Non-Allergen | Non-Toxic | HLA-A*11:01 | 21 | 30 | 10 | 154.39 | 0.88 | 100% |
| IMFMLIFNVK | (1.2092) Antigen | Non-Allergen | Non-Toxic | HLA-A*03:01 | 19 | 28 | 10 | 22.26 | 0.07 | 100% |
|  |  |  |  | HLA-A*02:01 | 19 | 28 | 10 | 31.18 | 0.28 |  |
|  |  |  |  | HLA-A*11:01 | 19 | 28 | 10 | 48.71 | 0.28 |  |
|  |  |  |  | HLA-A*31:01 | 19 | 28 | 10 | 110.67 | 1.2 |  |
|  |  |  |  | HLA-A*68:01 | 19 | 28 | 10 | 157.69 | 1.1 |  |
| MFMLIFNVK | (1.1933) Antigen | Non-Allergen | Non-Toxic | HLA-A*31:01 | 20 | 28 | 9 | 77.36 | 0.8 | 100% |
|  |  |  |  | HLA-A*68:01 | 20 | 28 | 9 | 78.96 | 0.63 |  |
|  |  |  |  | HLA-A*33:01 | 20 | 28 | 9 | 109.68 | 0.43 |  |
|  |  |  |  | HLA-A*30:01 | 20 | 28 | 9 | 151.28 | 0.46 |  |
| MLIFNVKSK | (1.8488) Antigen | Non-Allergen | Non-Toxic | HLA-A*68:01 | 22 | 30 | 9 | 107.57 | 0.77 | 100% |
|  |  |  |  | HLA-A*03:01 | 22 | 30 | 9 | 134.61 | 0.53 |  |
| MLIFNVKSKL | (1.3461) Antigen | Non-Allergen | Non-Toxic | HLA-A*02:03 | 22 | 31 | 10 | 39.28 | 0.62 | 100% |
| RIGTVAAKR | (1.7688) Antigen | Non-Allergen | Non-Toxic | HLA-A*31:01 | 49 | 57 | 9 | 50.9 | 0.55 | 100% |
| RIGTVAAKRY | (1.4405) Antigen | Non-Allergen | Non-Toxic | HLA-A*30:02 | 49 | 58 | 10 | 25.62 | 0.04 | 100% |
| WSRIGTVAAK | (1.6302) Antigen | Non-Allergen | Non-Toxic | HLA-A*68:01 | 47 | 56 | 10 | 171.78 | 1.2 | 100% |
