# Supplementary material for: Multi-epitope vaccine design against Monkeypox virus: An immunoinformatics approach
Source: PLoS One. 2026 Feb 13;21(2):e0342087. doi: 10.1371/journal.pone.0342087 (PMC12904572; doi:10.1371/journal.pone.0342087)
Supplement: S2 Table — (DOCX) [file pone.0342087.s014.docx]

| peptide | Antigenicity, Allergenicity | Allele | Start | end | length | core_peptide | ic50 | rank | Conservancy % |
| --- | --- | --- | --- | --- | --- | --- | --- | --- | --- |
|  | Toxicity |  |  |  |  |  |  |  |  |
| GFYFEIARIENEMKI | (0.5894) Antigen | HLA-DRB1*11:01 | 1 | 15 | 15 | YFEIARIEN | 45.7 | 4.7 | 100% |
|  |  | HLA-DRB1*04:05 | 1 | 15 | 15 | FEIARIENE | 63.3 | 2.6 |  |
|  |  | HLA-DPA1*03:01/DPB1*04:02 | 1 | 15 | 15 | YFEIARIEN | 84.3 | 3.5 |  |
|  |  | HLA-DRB5*01:01 | 1 | 15 | 15 | IARIENEMK | 130.3 | 15 |  |
|  |  | HLA-DRB1*01:01 | 1 | 15 | 15 | FYFEIARIE | 135.1 | 31 |  |
|  |  | HLA-DPA1*02:01/DPB1*01:01 | 1 | 15 | 15 | YFEIARIEN | 139 | 3 |  |
| SFFGLFDINVIGLIV | (0.8092) Antigen | HLA-DRB1*13:02 | 1 | 15 | 15 | FDINVIGLI | 20.6 | 2.9 | 100% |
|  |  | HLA-DRB1*01:01 | 1 | 15 | 15 | FFGLFDINV | 36.6 | 13 |  |
|  |  | HLA-DQA1*01:01/DQB1*05:01 | 1 | 15 | 15 | FFGLFDINV | 64.6 | 0.97 |  |
|  |  | HLA-DRB1*07:01 | 1 | 15 | 15 | FGLFDINVI | 71.2 | 8.3 |  |
|  |  | HLA-DRB3*01:01 | 1 | 15 | 15 | FDINVIGLI | 155.3 | 5.3 |  |
|  |  |  |  |  |  |  |  |  |  |
|  |  | HLA-DRB3*02:02 | 1 | 15 | 15 | FDINVIGLI | 177.9 | 6.1 |  |
