# Supplementary material for: Multi-epitope vaccine design against Monkeypox virus: An immunoinformatics approach
Source: PLoS One. 2026 Feb 13;21(2):e0342087. doi: 10.1371/journal.pone.0342087 (PMC12904572; doi:10.1371/journal.pone.0342087)
Supplement: S3 Table — (DOCX) [file pone.0342087.s015.docx]

| Model | GDT-HA | RMSD | MolProbity | Clash score | Poor rotamers | Rama favored |
| --- | --- | --- | --- | --- | --- | --- |
| Initial | 1.0000 | 0.000 | 0.793 | 1.0 | 0.0 | 99.6 |
| MODEL 1 | 0.9676 | 0.350 | 1.222 | 4.5 | 0.5 | 99.6 |
| MODEL 2 | 0.9686 | 0.346 | 1.252 | 4.8 | 0.8 | 99.6 |
| MODEL 3 | 0.9717 | 0.342 | 1.211 | 4.3 | 0.3 | 99.6 |
| MODEL 4 | 0.9769 | 0.338 | 1.128 | 3.4 | 0.5 | 99.6 |
| MODEL 5 | 0.9748 | 0.335 | 1.271 | 5.1 | 0.3 | 99.6 |
