# Supplementary material for: Multi-epitope vaccine design against Monkeypox virus: An immunoinformatics approach
Source: PLoS One. 2026 Feb 13;21(2):e0342087. doi: 10.1371/journal.pone.0342087 (PMC12904572; doi:10.1371/journal.pone.0342087)
Supplement: Supplementary file 5 [file pone.0342087.s008.docx]

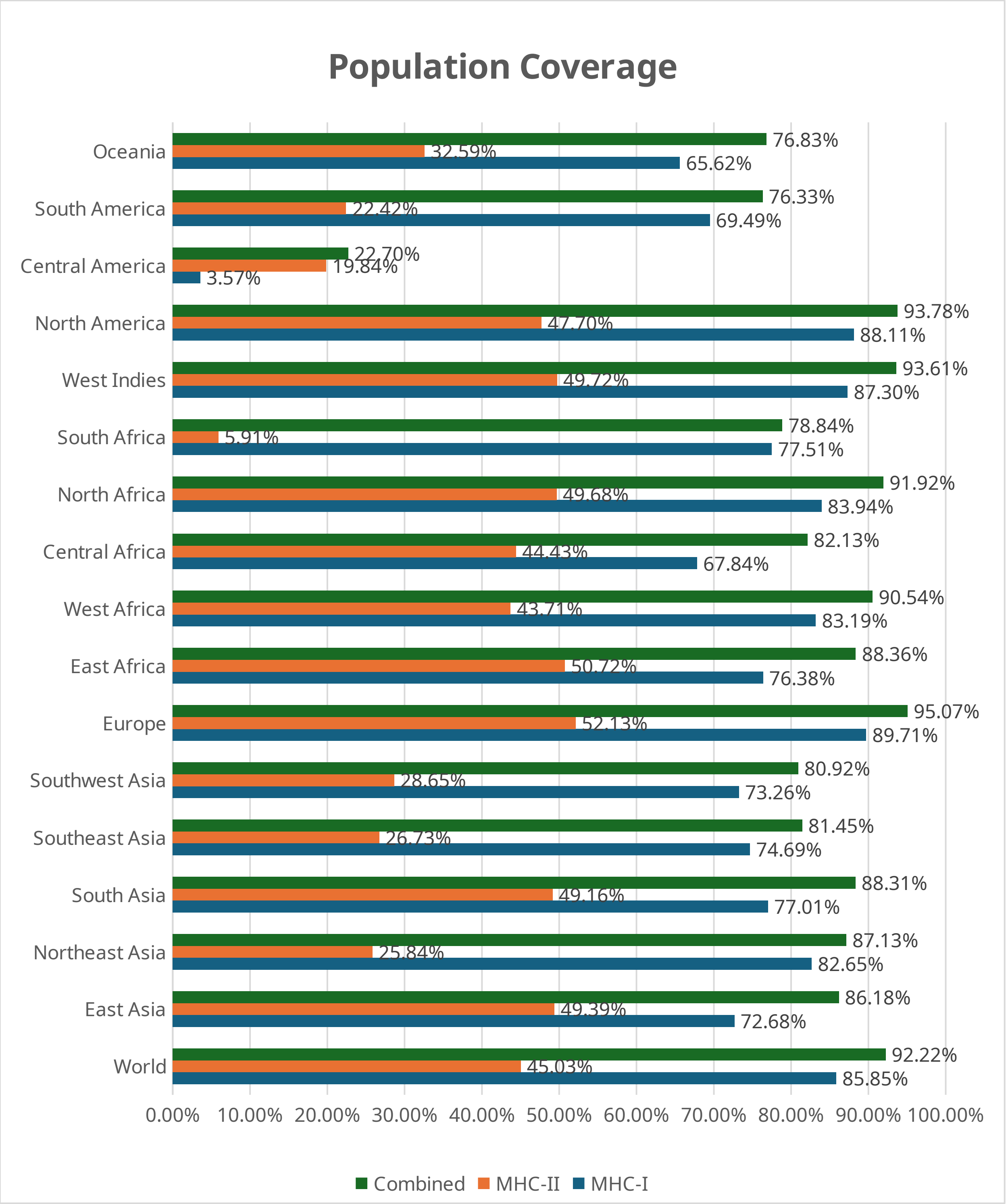
Population Coverage
Oceania
76.83%
32.59%
65.62%
South America
76.33%
22.42%
69.49%
Central America
22.70%
19.84%
3.57%
North America
93.78%
47.70%
88.11%
West Indies
93.61%
49.72%
87.30%
South Africa
78.84%
5.91%
77.51%
North Africa
91.92%
49.68%
83.94%
Central Africa
82.13%
44.43%
67.84%
West Africa
90.54%
43.71%
83.19%
East Africa
88.36%
50.72%
76.38%
Europe
95.07%
52.13%
89.71%
Southwest Asia
80.92%
28.65%
73.26%
Southeast Asia
81.45%
26.73%
74.69%
South Asia
88.31%
49.16%
77.01%
Northeast Asia
87.13%
25.84%
82.65%
East Asia
86.18%
49.39%
72.68%
World
92.22%
45.03%
85.85%
0.00% 10.00% 20.00% 30.00% 40.00% 50.00% 60.00% 70.00% 80.00% 90.00% 100.00%
Combined MHC-II MHC-I
